# Simple Porifera holobiont reveals complex interactions between the host, an archaeon, a bacterium, and a phage

**DOI:** 10.1093/ismejo/wrae197

**Published:** 2024-10-07

**Authors:** Alessandro N Garritano, Zhelun Zhang, Yunke Jia, Michelle A Allen, Lilian J Hill, Unnikrishnan Kuzhiumparambil, Cora Hinkley, Jean-Baptiste Raina, Raquel S Peixoto, Torsten Thomas

**Affiliations:** Centre for Marine Science and Innovation, School of Biological, Earth and Environmental Sciences, Faculty of Science, The University of New South Wales, Kensington, NSW 2052, Australia; Centre for Marine Science and Innovation, School of Biological, Earth and Environmental Sciences, Faculty of Science, The University of New South Wales, Kensington, NSW 2052, Australia; Centre for Marine Science and Innovation, School of Biological, Earth and Environmental Sciences, Faculty of Science, The University of New South Wales, Kensington, NSW 2052, Australia; Centre for Marine Science and Innovation, School of Biological, Earth and Environmental Sciences, Faculty of Science, The University of New South Wales, Kensington, NSW 2052, Australia; Universidade Federal do Rio de Janeiro, Instituto de Biologia, Departamento de Microbiologia Paulo de Goes, LEMM Laboratory, 21941-902, Rio de Janeiro, Brazil; Climate Change Cluster, University of Technology Sydney, Broadway, New South Wales 2007, Australia; Climate Change Cluster, University of Technology Sydney, Broadway, New South Wales 2007, Australia; Climate Change Cluster, University of Technology Sydney, Broadway, New South Wales 2007, Australia; Division of Biological and Environmental Science and Engineering (BESE), King Abdullah University of Science and Technology, Biological and Environmental Science and Engineering Division, Thuwal 23955 – 6900, Saudi Arabia; Centre for Marine Science and Innovation, School of Biological, Earth and Environmental Sciences, Faculty of Science, The University of New South Wales, Kensington, NSW 2052, Australia

**Keywords:** hexactinellid, symbiosis, flux-balance analysis, metagenome-assembled genomes, metatranscriptomics, metabolic interactions

## Abstract

The basal metazoan phylum Porifera (sponges) is increasingly used as a model to investigate ecological and evolutionary features of microbe–animal symbioses. However, sponges often host complex microbiomes, which has hampered our understanding of their interactions with their microbial symbionts. Here, we describe the discovery and characterization of the simplest sponge holobiont reported to date, consisting of the deep-sea glass sponge *Aphrocallistes beatrix* and two newly-described microbial symbionts: an autotrophic ammonia-oxidizing archaeon and a bacterial heterotroph. Omics analyses and metabolic modeling revealed the dependency of the ammonia-oxidizing archaea on sponge-derived ammonia to drive primary production, which in turn supports the bacterium’s growth by providing the dicarboxylate fumarate. Furthermore, virus-mediated archaeal lysis appears crucial to overcome the bacterium’s vitamin B_12_ auxotrophy. These findings reveal that the exchanges of vitamin B_12_ and dicarboxylate may be evolutionarily conserved features of symbiosis as they can also be found in interactions between free-living marine bacteria, and between microbes and plants or diatoms.

## Graphical Abstract

**Graphical Abstract:**
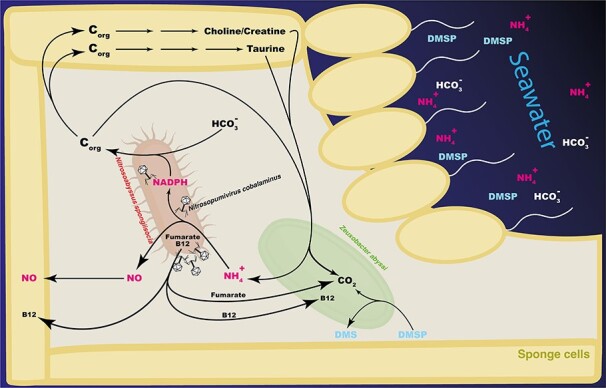

## Introduction

Sponges (Porifera) are among the most ancient extant metazoan phyla [1] and are widely distributed in aquatic and marine habitats around the globe [2]. They arguably represent one of the most ancient occurrences of symbiotic interactions between microorganisms and metazoans [3–5], and many of the microbial interactions described so far in sponges have broad analogs in other metazoans. For example, photosymbionts can transfer organic carbon to some sponge species [6] and in many corals [7], symbionts can provide chemical defense to some sponges [8] and some insect species [9], and symbionts can degrade complex carbohydrates in many sponges [10] and also in the mammalian gastrointestinal tract [11]. Thus, sponge holobionts are considered valuable model systems to characterize ancient or conserved ecological and evolutionary features of microbe–metazoan symbiosis [12].

Sponges often harbor diverse microbial communities, containing dozens or hundreds of genetically distinct bacterial and archaeal symbionts [13]. Studies have explored the potential of microbes to fix carbon and nitrogen [14, 15], to perform nitrification and denitrification [16], contribute to sulfur and phosphorus metabolism [17, 18] or process dissolved or particulate organic matter [19] at the level of individual symbiont types or the whole community. However, efforts to understand interactions within the entire symbiont community and how this might integrate into the host’s metabolism are uncommon due to the complexity of the community and metabolic processes. Hence, simpler systems, such as the shallow-water *Ianthella basta* and its three bacterial and archaeal symbionts [15], have been previously studied to better understand interactions within sponge holobionts.

Previously, our group reported the microbial composition of different deep-sea sponge species in the South Atlantic based on 16S ribosomal ribonucleic acid (rRNA) gene sequencing and this indicated an extremely simple microbiome for the hexactinellid (glass) sponge *Aphrocallistes beatrix*, which was distinct from those of the surrounding seawater or nearby sediments [20]. Here, we show that *A. beatrix* has apparently only two unicellular symbionts, making it the simplest Porifera holobiont discovered so far, and provide an extensive analysis of the organismal interactions. We generated complete metagenome-assembled genomes (MAGs) for its archaeal and bacterial symbionts and used metatranscriptomics, metabolite quantification, and metabolic modeling to reveal the crucial role of vitamin B_12_ and fumarate in the maintenance of the symbiosis. Finally, we also show that an archaeal virus can play an important role in the dynamics of this symbiosis by abating the bacterium’s vitamin B_12_ auxotrophy.

## Materials and methods

### Sample collection and site

*A. beatrix* specimens were collected using a remotely operated vehicle according to previously described methodology [20] at a depth of ~700 m at two different sites (sampling sites 1 and 5, Supplementary Table 1) during an expedition in the Campos Basin (22°38’S/40°25’W), near Rio de Janeiro, Brazil, in August - September 2021. The sites were characterized by sediment interspersed by corals, sponges and hard-substratum rubble. Sampling was performed with a grabber and specimens were placed into individual containers to minimize (cross-)contaminations. Sponge specimens were identified as previously described [20].

### Metagenomic analysis

Microbial cells were enriched from the sponge tissue using a modification of a previous protocol [21]. Briefly, ~20 g of *A. beatrix* tissue were homogenized using a bench blender (Hamilton Beach, 250 mL triton cup, Glen Allen, VA, USA) in 20 mL of calcium- and magnesium-free seawater (CMFSW). The homogenate was filtered through a sterile 100 μm filter (MF-Millipore, Carrigtwohill, Ireland) and centrifuged twice for 15 min at 300 *g* and 4°C. The supernatant was filtered twice through 20 μm filters (MF-Millipore), followed by a filtration through a 3 μm filter (MF-Millipore) and then centrifuged for 15 min at 15000 *g* and 4°C. The supernatant was discarded, and the cell pellet was resuspended in 2 mL of sterile CMFSW. Deoxyribonucleic acid (DNA) from the cell pellet was extracted using the DNeasy PowerSoil Pro Kit (Qiagen, Hilden, Germany) according to the manufacturer’s instruction, except for the FastPrep (MP Biomedicals, Irvine, CA, USA) step, where an intensity 4 for 30 s was used. The DNA obtained was quantified using a Qubit 3 (Thermo Fisher Scientific, Waltham, MA, USA) assessed for purity on a Nanodrop 1000 (Thermo Fisher Scientific) and analyzed on a TapeStation (Agilent, Santa Clara, CA, USA).

Library preparation (ligation sequencing genomic DNA—native barcoding SQK-NBD112.96 kit) and DNA sequencing were performed on a PromethION sequencer (Oxford Nanopore Technologies—ONT, Oxford, UK) using a flow cell FLO-PRO112 with R.10.4 chemistry at the KCCG Sequencing Laboratory (The Garvan Institute of Medical Research, Sydney, Australia). Basecalling was done using Guppy (v6.1.5—ONT) with the high accuracy model and assembled using Flye (v.2.9.0, parameters: —nano-raw and —meta) [22]. The assembly was polished using Medaka (ONT) and reads were mapped back to the polished assembly with Bowtie2 (v2.4.5) [23] to assess assembly quality and sequencing depth. MAGs were binned from the assembly using Metabat2 with default settings. The bins derived from the metagenomic assembly graph were improved using iterative read mapping and reassembly. GTDK-Tk (v.2.1.0) [24] based on the Genome Taxonomy Database (GTDB, http://gtdb.ecogenomic.org) release 214 was used to assign taxonomy to each MAG using the de novo workflow. Functional annotation of the MAGs was performed by running similarity searches with Diamond v2.1.4 against the NCBI non-redundant protein database (release 220) [25], Pfam (release 35)[26], TIGRFAM [27], and the Uniref databases (release 032023) [28].

Viral and proviral sequences were identified and taxonomically classified using geNomad v.1.7.4 [29]. Viral genomes were dereplicated based on pairwise average nucleotide identity (ANI) of 95% and checked for quality and completeness using CheckV v.1.0.1 [30]. Coverage was calculated by mapping the raw reads to the viral contigs using Bowtie2 v.2.4.2 [23] and Samtools v.1.15.1 [31]. MMseqs2 (release 15-6f452) [32] was used to annotate the viral genomes against PHROGS [33], PFAM [26] (release 35), KEGG [34], and COG [35].

### Ribonucleic acid extraction and gene expression analysis

Total RNA was extracted from 0.5 g of six frozen *A. beatrix* specimens with Trizol and bead-beating, and then purified with a PureLink RNA Mini kit (Thermo Fisher Scientific). rRNA was depleted using the RiboMinus kit with a mix of Eukaryote and Prokaryote probes (Thermo Fisher Scientific) according to manufacturer’s instructions, followed by NextSeq library construction (Illumina, San Diego, CA, USA) . Sequencing was conducted on a NextSeq System (Illumina) with 150 bp paired-end chemistry on a MID flow-cell at the Ramaciotti Centre for Genomics (UNSW, Australia) using manufacturer's instructions.

Paired-end reads were merged and quality-filtered using PEAR (v.0.9.11) [36]. SortMeRNA (v.4.3.3) [37] was used to remove rRNA reads, and the remaining reads were mapped back against the MAGs using Bowtie2 (v.2.4.5) [23]. Expression of the coding sequences for each MAG was estimated with HTSeq (v.2.0.2) [38]. Data were normalized using DeSeq2 [39] and expressed as normalized counts.

### Fluorescent *in situ* hybridization and scanning electron microscopy

Samples for fluorescence *in situ* hybridization (FISH) were fixed immediately after collection in 2% paraformaldehyde overnight at 4°C, rinsed in phosphate buffered saline (PBS) for 5 min and kept in a solution of PBS:ethanol (EtOH) (1:1, v:v) at 4°C. Samples were washed with PBS and then incubated in a 15% sucrose solution for 3 h at 4°C, followed by a substitution with optical coherence tomography (OCT) medium (Tissue Freezing Medium, OCT compound, Leica Biosystems, Wetzlar, Germany). For this, samples were incubated at 4°C with 15% sucrose/OCT buffers with increasing concentrations of OCT (35:10, overnight; 15:10 for 6 h; 15:30 overnight and 100% OCT for 4 h). Samples were transferred to disposable base molds (Leica Biosystems), frozen at −80°C overnight, then sectioned to 8 μm thickness with an Epredia CryoStar NX70 Cryostat (Leica Biosystems) and finally placed onto adhesion microscope slides (Menzel Gläser, SuperFrost Plus, Thermo Fisher Scientific).

Sponge tissue was dehydrated in an aqueous ethanol series (50%, 80%*,* and 96%) each for 5 min and then air-dried. The CREN499 probe (5′-[Cy5]-CTGGTGTCAGCCGCCG-3′) was used to target archaeal cells (i.e. *Nitrosoabyssus spongiisocia*) and the EUB338 probe (5′-[Atto-488]-GCTGCCTCCCGTAGGAGT-3′) was simultaneously used for bacteria (i.e. *Zeuxoniibacter abyssi*) [40, 41]. A non-sense non-EUB (5′-[Cy5]-ACTCCTACGGGAGGCAGC-3′) probe was applied as a control to check for unspecific binding to *A. beatrix* samples and no signal was ever observed (Supplementary Fig. 10). Hybridization was performed for 1.5 h at 46°C in hybridization buffer (900 mM NaCl, 20 mM Tris–HCl pH 7.4, 0.01% sodium dodecyl sulfate (SDS), 35% formamide) containing 8 ng/μl of each probe (Biomers, Ulm, Germany). Slides were then washed with a pre-warmed washing buffer (90 mM NaCl, 0.2 M Tris–HCl pH 7.4, 5 mM ethylenediaminetetraacetic acid (EDTA), 0.01% SDS) and incubated for 30 min at 48°C in the washing buffer. The slides were briefly washed with ice-cold distilled water, incubated with 1 ng/μl 4′,6-diamidino-2-phenylindole (DAPI) for 10 min at room temperature, rinsed with ice-cold distilled water, air dried, and mounted with antifade mountant (ProLong Diamond Antifade Mountant, Thermo Fisher Scientific). Images were acquired using a Zeiss LSM 900 upright confocal microscope with an Airyscan 2 detector at a resolution of 50 nm. Images were analyzed using Fiji [42] and we considered microbial cells between 0.8 and 2 μm in length. For quantitative FISH, we counted the cells in four fields of views (201.64 × 201.64 μm) of three different sponge individuals. For the Z-stack analysis, a region of 100.57 × 100.57 × 3.9 μm was scanned and 17 pictures were taken (step depth 250 nm) and the cell count results were extrapolated to the volume of 1 cm^3^.

Samples for scanning electron microscopy (SEM) were fixed immediately after collection in a solution of 2.5% glutaraldehyde + 4% paraformaldehyde + 0.1 M sodium cacodylate buffer overnight at 4°C, washed 3 × 10 min in sodium cacodylate buffer and kept in the same buffer at 4°C. The pre-fixed samples were cut into 1 × 1 × 2 mm pieces, rinsed with fresh 2.5% glutaraldehyde in 0.1 M sodium cacodylate buffer (pH 7.2) and processed using a microwave tissue processing system according to manufacturer’s instructions (PELCO BioWave Pro+, Ted Pella, Redding, CA, USA). Samples were stained with 1% osmium tetroxide solution in 0.1 M sodium cacodylate buffer, washed with 0.1 M sodium cacodylate buffer and double-distilled water (ddH_2_O) and subsequently dehydrated through a series of ethanol washes (30%, 50%, 70%, 80%, 90%, 95%; 100% × 3). The dehydrated samples were infiltrated with increasing concentrations of LR white resin (LR-white/ethanol 1:2, 2:1, 100% LR-white × 3, Sigma-Aldrich, Saint Louis, MI, USA). The specimens were then transferred into a sample embedding mold (Sigma-Aldrich) and polymerized at 55°C for 48 h. Blocks were sectioned to 100 nm thickness using an EM UC7 ultramicrotome (Leica Biosystems) equipped with an ultra 35° Jumbo diamond knife (Diatome AG, Nidau, Switzerland) onto adhesion microscope slides (Menzel Gläser, SuperFrost Plus, Thermo Fisher Scientific). The slides were dried at 50°C for at least 20 min to enhance adhesion. The slides were stained with 2.5% uranyl acetate in 100% ethanol for 15 min at room temperature, followed by 3% lead citrate (w/v) staining for 10 min in a chamber with sodium hydroxide pellets. After washing with ddH_2_O and air drying, samples were carbon-coated using an Emitech K575X Sputter Coater (Quorum Technologies, Lewes, UK) before imaging. SEM images were acquired with an FEI Nova NanoSEM 450 (FEI Company, Hillsboro, Oregon, USA) at 5 kV with a circular backscatter detector.

### Metabolite extraction and ultra-high-pressure liquid chromatography coupled to mass spectrometry

Six *A. beatrix* samples were thawed and dried with Kimwipes (Kimberly-Clark, Texas, USA) to remove frozen water droplets. The sample volume was determined, then weighted and homogenized in 10 mL of methanol, followed by a 30 min sonication in an ice bath and subsequent centrifugation. The supernatant was dried in a vacuum concentrator (Concentrator Plus, Eppendorf, Hamburg, Germany) at 30°C and stored in a clean plastic bag with desiccants until further analysis. Dried extracts were reconstituted in MilliQ water and syringe-filtered (0.2 μm Nalgene Sterile Syringe Filters, Thermo Fisher Scientific).

The ultra high performance liquid chromatography coupled to a mass spectrometer (UHPLC–MS) system consisted of a Nexera MP UHPLC interfaced to a LCMS-8060 triple quadrupole mass spectrometer equipped with an electrospray ionization source (Shimadzu, Kyoto, Japan). A UPLC BEH (Ethylene Bridged Hybrid) HILIC (Hydrophilic Interaction Liquid Chromatography) column (2.1 × 100 mm, 1.7 μm, Waters Acquity, Milford, MA, USA) was used for the on-line separation. Metabolites were separated isocratically at a flow rate of 0.25 ml min^−1^ with the mobile phase consisting of 70% acetonitrile and 30% MilliQ water for a total run time of 10 min. The injection volume was 2 μl, with the following mass spectrometry acquisition parameters: positive ion mode; heating and drying gas flow: 10 L min^−1^; nebulizing gas flow: 2 L min^−1^; the interface, desolvation line, heating block and column oven temperatures of 300°C, 250°C, 400°C, and 30°C, respectively. Acquisition was done in multiple reaction monitoring mode with transitions optimized using the LabSolutions software and acquisition parameters are reported in Supplementary Table 2. Quantification of analytes were achieved with calibration curves established using standards of betaine, choline, creatine, dimethylsulfoniopropionate (DMSP), and taurine that ranged in concentration from 1 ppb to 200 ppb.

### Metabolic modeling

Metabolic models were generated with the program gapseq, which has been shown to outperform other automated modeling tools in terms of prediction accuracy [43]. MAGs were searched against the UniProt database [44] and the Transporter Classification Database (TCDB) [45] using tblastn implemented in the *gapseq -find* module with thresholds for bit scores >100 and coverages >70. Drafts of genome-scale metabolic models were constructed using the *gapseq -draft* module based on mapped enzyme reactions. Missing reactions were filled in during the *gapseq -fill* step using a growth medium-defined based on transporters found to be expressed in the transcriptomic data and the UPLC–MS results. Filled reactions were manually curated for each of the MAGs by considering the literature on related reference organisms (Supplementary Table 3).

The biomass formula for *N. spongiisocia* was based on the archaeon *Methanosarcina barkeri* [46] with the cofactors tetrahydrosarcinapterin, F430, co-enzyme M, cytochromes F420–2, F390-A*,* and F390-G, HTP involved in the methanogenesis being removed. The 3HP/4HB cycle (BioCyc ID: PWY-5789), which was expressed in the transcriptomic data but not detected in the *gapseq -find* step, was manually added to the model of *N. spongiisocia* (Supplementary Table 3). The auto-filled reaction BioCyc ID: 2.1.3.1-RXN catalyzed by an S-methylmalonyl-CoA carboxyl transferase was turned off to prevent propanoyl-CoA from being deviated from the 3HP/4HB cycle, as this enzyme was not found in the *N. spongiisocia* genome. As the whole anaerobic vitamin B_12_ biosynthesis pathway was expressed, calomide was added to the biomass formula to replace the vitamin B_12_ derivative adenosyl factor III used by gapseq. The biosynthesis of tetrahydrofolate was also manually filled in, as the key enzyme dihydropteroate synthase was found to be expressed.

A draft model for *Z. abyssi* was developed using the general biomass formula of *Escherichia coli*. As 13 out of the 15 reactions required for the biosynthesis of lipid-A were autofilled in and because marine *Gammaproteobacteria* are known to have a distinct set of lipopolysaccharides from other *Gammaproteobacteria* [47]*,* lipid-A was removed from the biomass formula. A BCCT- type transporter was found in the *Z. abyssi* MAG and this class of transporters has previously been shown to take up different betaine-like molecules, including carnitine, choline, and DMSP [48]. The transcriptomic data further showed active pathways for the degradation of creatine, DMSP, choline, and taurine, and therefore specific import reactions for these compounds were added into the model. The reaction for propionate-CoA ligase (RXN-21753), which catalyzes the activation of acrylate during DMSP degradation, was manually added into the model, as it is not in the gapseq database. Additional modifications of reactions are explained in Supplementary Table 3.

BacArena v 1.8.2 [49] was used to simulate the individual growth and evaluate the metabolic interactions between symbionts. BacArena is a community modeling software that simulates interactions and cross-feeding dynamics between microorganisms and with the surrounding environment (medium) using flux balance analysis (FBA). The metabolic models for *N. spongiisocia* and *Z. abyssi* were placed into an arena measuring 12 × 12 mm with 1600 grids, accommodating one cell per grid (each grid size is equivalent to 9 × 10^−8^ cm^2^). Initially, 95% of the grid units were occupied, with the abundance of each organism reflecting their natural abundance measured by genome coverage in the metagenome of *A. beatrix*. The simulation was allowed to reach stable states by randomly removing 2% of cells in every cycle. Cell death was varied and the biomass equivalent to the lysed cells was made available for other cells in the area to utilize. The *in silico* growth media was based on previous literature on the composition of inorganic compounds in seawater [50–53], except oxygen concentrations, which were measured during the expedition (Supplementary Table 4). The concentration of the organic compounds was determined based on the UHPLC results (see above).

## Results

### *Aphrocallistes beatrix* hosts a microbiome dominated by two symbionts

Our previous 16S rRNA gene analysis revealed that the microbiome of *A. beatrix* is very simple and consistently dominated (i.e. relative read abundance > 1%) by one archaeal and one bacterial amplicon sequence variant, with the former making up 97.70 ± 0.01% of the total read abundance [20]. To further characterize this microbiome, we performed long-read metagenome sequencing of two *A. beatrix* samples (S29 and S84) and retrieved MAGs for only one archaeon and one bacterium in each sample. The archaeal MAG recruited 94.6% and 66.1% of all binned reads in samples S29 and S84, respectively, while the bacterial MAG recruited 4.5% and 32.5% of reads, respectively.

**Figure 1:**
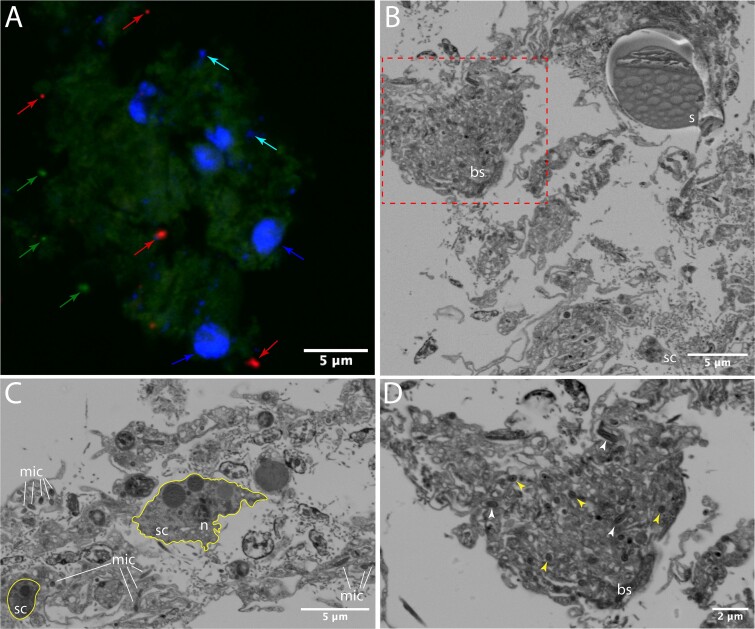
FISH and SEM images of the deep-sea sponge *A. beatrix* highlighting microbial symbionts and the sponge’s structure. (A) Representative FISH image of a section of *A. beatrix* showing archaeal cells (red; red arrows) and bacterial cells (bright green; green arrows). DAPI-stained sponge nuclei (blue arrows) and putative mitochondria (cyan arrows) are shown in the pale green autofluorescence of the sponge tissue. Further images are in the Supplementary Fig. 1. (B) Scanning electron micrography of an overview of *A. beatrix* tissue (s = spicule, bs = bacteriosyncytia-like structure, sc = sponge cell). C: Scanning electron micrography of a different *A. beatrix* section highlighting an sc, outlined in yellow, its nucleus (n) and microbial cells (mic) distributed within an extracellular matrix, probably the sponge mesohyl. (D) Magnified section of dashed red rectangle in Fig. 1B highlighting the bs and the microbial symbionts. Most of the cells seem to have the same rod-shaped morphology (white arrows) and measure between 0.72–2 μm long and 0.35 μm wide. The ovoid shapes (yellow arrows) observed in the images are likely caused by the sectioning plane.

FISH analysis revealed that the archaeal and bacterial cells are spread across the *A. beatrix* tissue with average relative abundances of 88.8 ± 3.3% for the archaeal probe and 11.2 ± 3.3% for the bacterial proble (Fig. 1, Supplementary Fig. 1). A ratio of 22.4 ± 13.4 bacterial/archaeal cells per sponge nuclei was observed (Supplementary Fig. 1), which falls within the range previously described for high microbial abundance sponges [54]. Volumetric analysis of z-stack images (Supplementary Video 1) further estimated a total bacterial and archaeal abundance of 8.6 × 10^8^ cells/cm^3^ sponge tissue. Scanning electron microscopy further revealed symbionts localized in an extracellular matrix (Fig. 1B and C), with occasionally dense aggregates of rod-shaped cells embedded in an amorphous structure (Fig. 1D), which is reminiscent of the bacteriosyncytia recently described in the hexactinellid *Vazella pourtalesii* [55].

The archaeal MAGs retrieved from samples S29 and S84 had an ANI of 98.99% and no differences in gene content. The bacterial MAGs from the two samples had an ANI of 94.46% and gene analysis revealed 62 transposons in the former and 20 in the latter, but no other genomic differences. While we could not identify any gene disruption through the transposons, their differential abundance between the bacterial populations in the two sponge specimens suggests an ongoing process of genomic evolution, which might be related to adaptation to a specific host environment [56]. The MAGs obtained from sample S29 were closed and circular and were thus used for further analysis.

Maximum-likelihood phylogenetic analysis of 53 marker protein sequences showed that the archaeal MAG belongs to the family *Nitrosopumilaceae* and forms a separate lineage from all other genera (Supplementary Fig. 2). The MAG had a maximum amino acid identity (AAI) of 62.68% when compared to the closest reference MAGs of this family (Supplementary Table 5) and had a relative evolutionary divergence value of 0.92 to the nearest taxon [57]. Therefore, we propose the name *Nitrosoabyssus spongiisocia* gen. nov., sp. nov., which has been endorsed by SeqCode [58] (etymology provided in supplementary information). Its genome has a size of 1.45 Mb, a GC content of 34.1% and is predicted to encode for 1682 protein-coding genes and one set of 16S, 23S*,* and 5S rRNA genes and 45 transfer RNA (tRNA) genes. *N. spongiisocia* possesses 11 genes encoding tetratricopeptide repeats, which belong to a class of eukaryotic-like proteins that potentially mediate molecular interactions between sponges and microorganisms [59]. The absence of genes encoding flagellin indicates that *N. spongiisocia* lacks motility. Three genes for restriction endonucleases were found and no clustered regularly interspaced short palindromic repeats (CRISPRs) were identified.

Phylogenetic analysis showed that the bacterial MAG belongs to the recently described family *Persebacteraceae* within the order *Tethybacterales* [60] in the class *Gammaproteobacteria* (Supplementary Fig. 3), with a maximum AAI of 60.90% when compared to all relevant reference MAGs (Supplementary Table 6). Therefore, this bacterium belongs to a new genus and we named it *Zeuxoniibacter abyssi* gen. nov., sp. nov., which has been endorsed by SeqCode [58] (etymology provided in supplementary information). Its genome has a size of 2.05 Mb and a GC content of 48.6%; and encodes 2327 predicted protein-coding genes, one set of 16S, 23S*,* and 5S rRNA genes and 41 tRNA genes. Three different restriction/modification systems were predicted in the genome, as well as four CRISPRs, alongside genes for four copies of the Cas1, two copies of the Cas2, and two copies of the Cas9 proteins.

### Metabolism of *Nitrosoabyssus spongiisocia* and *Zeuxoniibacter abyssi*

**Figure 2:**
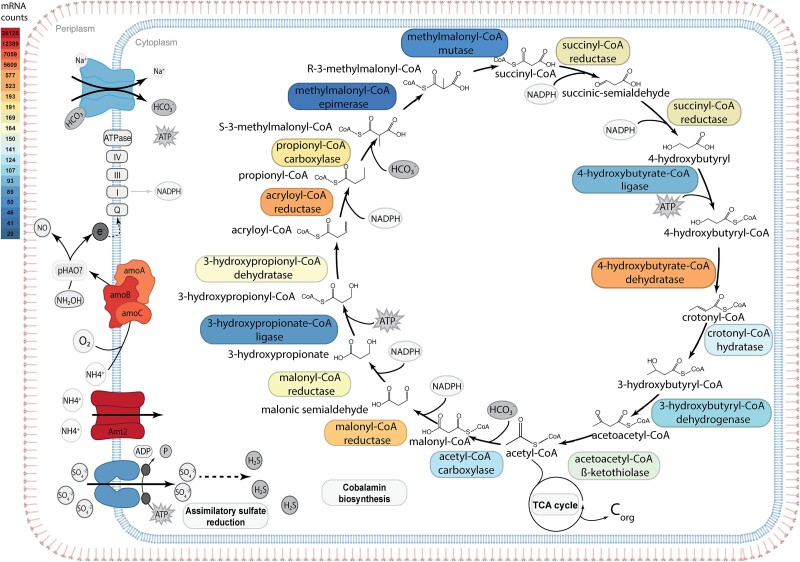
Metabolic reconstruction of *N. spongiisocia*. Amt2: high-affinity ammonia transporter. AmoABC: ammonia monooxygenase subunits A, B, and C; pHAO: putative hydroxylamine oxidase. S-layer is represented in pink and the cell membrane is represented in blue. Enzymes are color-coded according to their average expression levels.

Members of the *Nitrosopumilaceae* are typically chemolithoautotrophic and oxidize ammonium to nitric acid or nitrite to generate reductive power for carbon fixation [61]. Metatranscriptomic data for six different sponge replicates showed that all six genes for ammonia oxidation (*amoABCXYZ*) as well as *amt2* (encoding an ammonia permease) were among the most highly expressed ones (Fig. 2, Supplementary Tables 7 and 8). All genes for the 3-hydroxypropionate/4-hydroxybutyrate (3HP/4HB) cycle, which fixes carbon dioxide, were also expressed in *N. spongiisocia*, as well as genes for the assimilatory sulfate reduction, and cobalamin (vitamin B_12_) biosynthesis.

**Figure 3:**
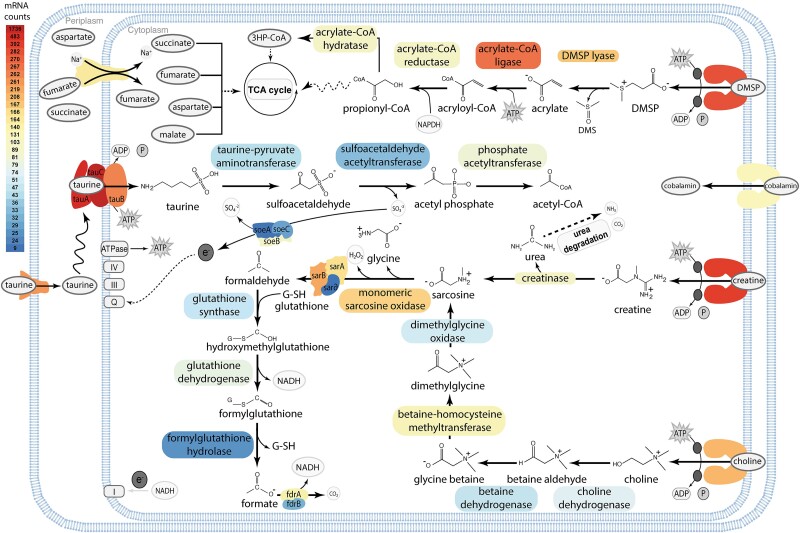
Metabolic reconstruction of Z. *abyssi* focusing on the potential energy sources. Enzymes are color-coded according to their average expression levels. SoeABC: sulfite oxidase subunits ABC; TauABC: taurine ABC transporter subunits ABC; SarABC: sarcosine oxidase subunits ABC; 3HP-CoA: 3-hydroxypropionate-CoA; fdrAB: formate dehydrogenase subunits AB.

The most highly expressed genes in *Z. abyssi* are related to taurine import and degradation using the taurine:2-oxoglutarate pathway to L-glutamate and sulfoacetaldehyde, with the latter ultimately being converted into acetyl-CoA and sulfite (Fig. 3, Supplementary Tables 9 and 10). All subunits of a sulfite dehydrogenase were found to be expressed, suggesting further oxidation of sulfite to sulfate, likely aiding in cell detoxification [62]. *Z. abyssi* also expressed transporters and catabolic enzymes for choline and creatine, which are both metabolized to sarcosine and then fully oxidized into CO_2_. Taurine, choline, and creatine are organic molecules commonly found in sponges [15, 63] and genes for their synthesis were detected by homology searches using reference sequences from the *Aphrocallistes vastus* genome against unbinned contigs of the *A. beatrix* metagenome (Supplementary Table 11) [64].

*Z. abyssi* expresses the genetic capacity to import and fully metabolize DMSP, an organic molecule not only known to be produced by coral-associated bacteria, phytoplankton, and algae [65, 66], but also appears to have a role in hydrostatic pressure protection in deep-sea sediment bacteria [67]. DMSP is likely metabolized through the expressed cleavage pathway, which produces dimethyl sulfide and acrylate, with the latter ultimately being converted to acetyl-CoA. Finally, *Z. abyssi* also expressed transporters for C4:dicarboxylate compounds, which can be metabolized through the tricarboxylic acid cycle [68].

While most of the genes involved in the degradation of these organic compounds were consistently expressed across the six transcriptomic datasets analyzed here, genes involved in the transport of C4:dicarboxylate compounds were not expressed in four samples (S44, S45, S70, and S71; Supplementary Fig. 4, Supplementary Table 10). Overall, only 76.4 ± 3.7% of all *Z. abyssi* genes were expressed in these four samples, compared to 96 ± 3.4% in the other two samples (S29 and S65). Several genes involved in the ribosome synthesis were downregulated in samples S44, S45, S70, and S71, which would suggest a lower translational activity [69]. Taken together, these difference in expression profiles suggest that *Z. abyssi* was taking up and utilizing different nutrients in a dynamic manner and we explored this further using metabolic modeling.

### Metabolic interactions between the two symbionts and the role of a novel virus

Aiming to understand the potential metabolic interactions between the two symbionts, we employed FBA based, genome-scale metabolic modeling in a spatio-temporal resolved simulation environment (see Material and methods for details). FBA suggests that *N. spongiisocia* can grow purely chemolithoautotrophically in seawater (Supplementary Fig. 5 and Supplementary Table 13) but requires ammonium concentrations that are higher than what we measured in the surrounding water at our deep-sea site (Supplementary Fig. 6, Supplementary Table 13). This indicates that *N. spongiisocia* requires additional ammonia from its host, which aligns with previous work that deep-sea hexactinellids release this compound [70].

As *Z. abyssi* had no genetic potential for autotrophy, we defined potential carbon sources by measuring and normalising (by sponge biomass in five different *A. beatrix* specimens) the abundance of DMSP, creatine, betaine, choline, and taurine (Supplementary Table 14), as their transporters and degradation pathways were highly expressed (see above). We then included these compounds at ecologically relevant concentrations into the *in silico* medium for the metabolic model of *Z. abyssi*, but found growth only when vitamin B_12_ was also provided (Supplementary Fig. 7; Supplementary Table 13). This vitamin B_12_ auxotrophy of *Z. abyssi* could potentially be abated by the biosynthetic capacity of *N. spongiisocia* (see above), but since there is no genetic evidence for an active vitamin B_12_ exporter, we postulated that transfer fluxes of this metabolite rely on the lysis of the archaeon.

**Figure 4:**
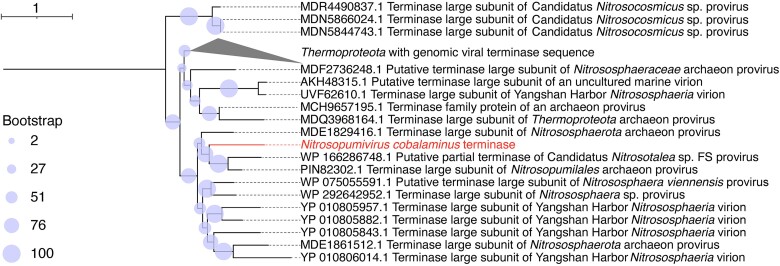
Phylogenetic analysis of terminase sequences of the virus *Nitrosopumivirus cobalaminus* (Aphro18_contig_2847 highlighted in red) and reference sequences in the NCBI Genbank database. Sequences were aligned using MUSCLE [70] and maximum-likelihood trees were constructed using IQTree v1.6.12 [71] (WAG model, nearest-neighbor interchanges) and 1000 bootstraps. The tree was rooted with a large terminase subunit sequence of *Harrekavirus harreka* (class *Caudoviricetes*, family *Aggregaviridae*—YP_010356946.1; not shown). Tree scale represents an average substitution of one amino acid in the terminase sequence.

To explore the potential mechanisms for lysis, we further analyzed the metagenome of *A. beatrix* and discovered a single, circular viral contig (Aphro18_contig_2847) of 43 Kbp length and encoding 61 proteins. The contig received very high read coverage in sample S84 (Supplementary Table 15). Phylogenetic analysis of this virus’ terminase protein sequence revealed a close association with *Thermoproteota* viruses in the class *Caudoviricetes*, including the formation of a monophyletic cluster with *Nitrososphaeria* viruses from the Yangshan Harbor, China (Fig. 4) [68]. These viruses have been shown to infect marine archaea, and most of them are predicted to infect *Nitrososphaeria*, the class that *N. spongiisocia* belongs to [68,69]. geNomad [26] failed to identify a sufficient number of marker genes for a genus, family or order level assignment of the viral contig. We therefore propose the name *Nitrosopumivirus cobalaminus* (nov. Order. *Iaravirales*, nov. Fam. *Anhangaviridae*, Genbank accession number PP848464, etymology provided in supplementary information) the virus that potentially infects *N. spongiisocia*.

**Figure 5:**
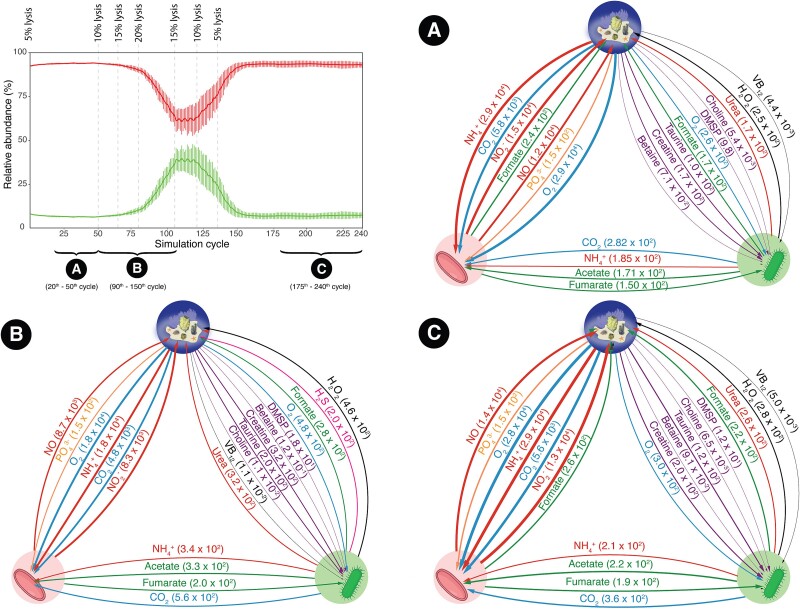
Population abundance and metabolic fluxes of the two-symbiont community model. The continuous lines and bars in the top left panel represent the relative abundance and its variation across replicate simulations for *N. spongiisocia* (red) and *Z. abyssi* (green) under different lysis rates (shown at top of graph). Predicted metabolic fluxes during the first (panel A), second (panel B), and third (panel C) stable metabolic flux profiles (see Supplementary Fig. 8) representing 99.12, 99.48, and 99.17% of the total fluxes, respectively. Width of arrows is proportional to the natural logarithm of fluxes and their values are given in brackets (fmol/h). Red cells at the bottom left of each panel represent *N. spongiisocia*, green cells at the bottom right represent *Z. abyssi*, and the top middle circle represents the sponge environment. The top 15 highest fluxes (excluding those for water and H^+^) plus those of betaine, choline, creatine, DMSP, and vitamin B_12_ are shown.

The discovery of the virus provided justification to apply a cellular lysis to *N. spongiisocia* in a two-symbiont metabolic model simulation. Based on the observations made above, the simulation was performed with a medium consisting of seawater supplemented with environmentally relevant concentrations of DMSP, creatine, betaine, choline, and taurine measured in the *A. beatrix* specimens, oxygen concentration observed in the deep-sea site (6.7 mg/L), and an ammonium concentration ~20 times higher than found in the deep-sea water. At a 5% lysis rate per simulation cycle, we found that *N. spongiisocia* and *Z. abyssi* could stably co-exist for at least 50 simulation cycles and at a ratio of ~95 to 5 cells, similar to what had been seen in sample S29 (see above and Fig. 5, top left panel). We then applied sequentially higher lysis rates to the community model and found that at a lysis rate of 20%, the population ratio of *N. spongiisocia* and *Z. abyssi* approached the one seen in sample S84 (Fig. 5, top left panel). This lysis value is similar to previous observations that archaeal viruses infecting *Thaumarchaeota* could lyse up to one-third of the total microbial biomass [75].

When applying these different lysis rates, we observed that the symbiont community reached two different stable states of metabolic fluxes (Fig. 5 top left panel, and Supplementary Fig. 8). At a basal 5% lysis rate (i.e. between simulation cycles 20 and 50), the metabolic flux profile was characterized by high environmental uptake of CO_2_, O_2_, and ammonium by *N. spongiisocia* (Fig. 5A and Supplementary Table 16). The resulting primary production by *N. spongiisocia* was predicted to lead to a release of fumarate and formate, with the former being used by *Z. abyssi* for cellular growth. The predicted uptake of fumarate by *Z. abyssi* is also consistent with the high expression of a porin that, based on a structural fold prediction, has an affinity for divalent anions, such as fumarate (Supplementary Figs 4 and 9 and Supplementary text).

The FBA also showed that at 5% archaeal lysis rate, the uptake fluxes of fumarate by *Z. abyssi* are similar to those of taurine and creatine (Fig. 5A, Supplementary Table 16), fifteen times higher than those of DMSP, as well as four and five orders of magnitude higher than those of betaine and choline, respectively. These order-of-magnitude differences are a consequence of the differences in environmental concentrations measured, but nevertheless indicate that metabolic cross-feeding of fumarate could represent a major energy and carbon source for *Z. abyssi*. The bacterium also releases CO_2_ and ammonium, which can be used by *N. spongiisocia*. The fluxes are however predicted to be one and two orders of magnitude smaller, respectively, than the fluxes from the environment, indicating that this metabolic interaction contributes little to the biomass and energy generation of *N. spongiisocia*. H_2_O_2_ is predicted to be released from the sarcosine oxidation by *Z. abyssi* (Figs 3 and 5). Several species of coral and sponges, including hexactinellids, have recently been recognized as “hotspots” for the release of reactive oxygen species (ROS) in the deep-sea, however the producers (i.e. host or symbionts) were not identified [76]. Our results indicate that symbiont metabolism could contribute to this ROS production.

In the second metabolic flux state, where the lysis rate progressively increased to 20%, uptake fluxes of ammonium and O_2_ decreased by ~1.5 times for *N. spongiisocia* and its lysis is predicted to result in ten-fold higher flux of vitamin B_12_ to *Z. abyssi*, which would abate its growth limitation (Fig. 5B, Supplementary Table 16). Indeed, fluxes of O_2_, taurine, and creatine from the environment into *Z. abyssi* increased by ~2-fold. The simulation also predicted that *Z. abyssi* has an increased production of acetate, which *N. spongiisocia* can use for a mixotrophic growth, similar to what has been previously demonstrated for some free-living marine ammonia-oxisiding archaea (AOA) [77]. Finally, when the lysis rate decreased again to 5%, metabolic fluxes mirror those of the first stable state (Fig. 5C, Supplementary Table 16). This revealed an inherent stability of the two-symbiont community model independent of prior lysis events.

## Discussion

Here, we present a detailed analysis of the interactions within the simplest sponge system found so far, which is comprised of the host *A. beatrix*, the archaeon *N. spongiisocia*, the bacterium *Z. abyssi*, and the virus *Nitrosopumivirus cobalaminus*.

*N. spongiisocia*, a chemolithoautotrophic AOA, dominates the symbiont community in *A. beatrix*, reaching relative abundances of up to 95%, as indicated by 16S rRNA gene sequencing, metagenomic read coverage, and quantitative microscopy analysis. It has previously been noted that AOA can make up a substantial proportion of the microbial community of various deep-sea sponges [20, 78], in contrast to many shallow-water sponges, where symbionts belonging to the *Pseudomonadota*, *Chloroflexota*, *Cyanobacteriota*, *Actinomycetota*, and *Acidobacteriota* are often dominant, and where photosynthetic microorganisms mainly underpin autotrophy [13, 79, 80]. How the primary production of AOA contributes to or interacts with other members of the sponge holobiont is however poorly understood.

Our data and analysis suggest that *N. spongiisocia* plays a vital role in the *A. beatrix* holobiont through the provision of reduced organic carbon compounds and vitamins. Specifically, while the genetic repertoire of *Z. abyssi* indicated its primary reliance on sponge-derived (e.g. taurine, creatine) or environmental nutrients (e.g. DMSP), quantification of these compounds and FBA-based metabolic modeling instead demonstrated the bacterium’s strong dependency on vitamin B_12_ and the dicarboxylate fumarate provided by *N. spongiisocia*. A dependence on a dicarboxylate has also been recently observed in the endosymbiosis between the N_2_-fixing rhizobia *Candidatus* Tectiglobus diatomicola and its diatom host *Haslea* sp. [81], suggesting that this compound class may play a widespread, fundamental role in the maintenance of symbiosis.

*Z. abyssi* belongs to the recently described sponge-specific taxon *Tethybacterales*, whose members appear to have entered symbiotic interactions with sponge species through several independent events [60, 82]. While the *Tethybacterales*’ overall variability in their genetic potential for carbon, nitrogen*,* and sulfur catabolism could contribute to a “scavenging lifestyle” based on the resources that are available in each specific sponge species [60, 83], growth of *Z. abyssi* in the *A. beatrix* holobiont is apparently also reliant on the metabolism of another symbiont.

The metabolic interactions between the two cellular symbionts appears to be also controlled by the action of a virus. While sponges have been noted to contain a range of viruses that could infect bacteria or archaea [84, 85], nothing is known about how (or if) they influence community dynamics. The inclusion of the newly discovered virus *N. cobalaminus* into our metabolic interaction model revealed that it may reduce the abundance of the dominant archaeon (i.e. killing-the-winner [86]), thus relieving some of the growth limitations (i.e. vitamin B_12_) experienced by *Z. abyssi*. Our model predictions thus resulted in an abundance dynamic of the two symbionts that could explain the variation in community structure seen in environmental samples (Fig. 5). A similar phenomenon has also recently been reported for a marine, free-living *Roseovarius* sp., which is lysed by a prophage causing a release of vitamin B_12_, which in turn promotes the growth of a co-occurring *Colwellia* sp. [87]. Viral lysis to abate vitamin B_12_ auxotrophy might thus be a shared feature of free-living and host-associated microbiomes.

The release of small organic molecules (e.g. fumarate, formate*,* and acetate) through the viral lysis of *N. spongiisocia* may not only impact the growth of the bacterial symbiont (Fig. 5), but could also represent a potential resource for the sponge, which can directly uptake dissolved organic matter [88]. It is also possible that the host directly consumes part of its symbiont population, as has been observed in some deep-sea sponges [89, 90], although for *A. beatrix* further work is required to ascertain this. Additional interactions with the host could involve nitric oxide derived from ammonia oxidation (Fig. 2), which can act as a signaling molecule for larval settlement and metamorphosis in sponges [91] as well as the production of ROS by *Z. abyssi*, which might aid the holobiont’s chemical defense against pathogens [76].

We did not observe any symbiont in the *A. beatrix* holobiont capable of oxidizing nitrite or to reduce it (or nitrate) to dinitrogen or ammonium, indicating the absence of nitrogen cycling. This apparently contrasts with shallow-water sponges, where nitrite or nitrate are often utilized by denitrifying symbionts [16, 92, 93]. This difference could be due to the deep-sea environment being depleted in organic carbon and possessing an excess of inorganic nitrogen [94–96]. In fact, water at our deep-sea locations had low organic C: inorganic N ratios (2.88 and 8.07) well below that of local surface water (112.01; Supplementary Table 1). Excess nitrogen in the filtering diet of *A. beatrix* would also explain a release of ammonium that the FBA required to support the growth *N. spongiisocia.*

The analysis of the simple *A. beatrix* holobiont illustrates how direct mutualistic interactions between the host and an AOA are combined with the actions of a parasitic virus and a bacterial scavenger. The interactions discovered here are also likely to play out in more complex sponge microbiomes and thus the information from the *A. beatrix* holobiont provides a foundation to model them in the future. Given the long evolutionary interactions between sponges and microbial symbionts, some of the principles discovered here might also occur in other metazoan symbiosis.

## Supplementary Material

Untracked_Supplementary_Information_V4_wrae197

Supplementary_Tables_V4_wrae197

Supplementary_video_1_wrae197

## Data Availability

All data are available in the main text or as supplementary materials. *N. spongiisocia* and *Z. abyssi* MAG assemblies are deposited under the accession numbers GCA_033471095.1 and GCA_033470255.1. The accession number for *Nitrosopumivirus cobalaminus* is PP848464. Metagenomic reads are deposited in the SRA under the accession number SRR25845006 and the metatranscriptomic dataset is available at the SRA under accession numbers SRR29297744–SRR29297749.
