# Supplementary material for: Simple Porifera holobiont reveals complex interactions between the host, an archaeon, a bacterium, and a phage
Source: ISME J. 2024 Oct 7;18(1):wrae197. doi: 10.1093/ismejo/wrae197 (PMC11525543; doi:10.1093/ismejo/wrae197)
Supplement: Untracked_Supplementary_Information_V4_wrae197 [file untracked_supplementary_information_v4_wrae197.docx]

**Supplementary Figures and Information for “Simple Porifera holobiont reveals complex interactions between the host, an archaeon, a bacterium, and a phage”**

Authors: Alessandro N. Garritano^1*^, Zhelun Zhang^1†^, Yunke Jia^1†^, Michelle A. Allen^1^, Lilian Hill^3^, Unnikrishnan Kuzhiumparambil^2^, Cora Hinkley^2^, Jean-Baptiste Raina^2^, Raquel Peixoto^4^, Torsten Thomas^1*^

1 – Centre for Marine Science and Innovation, School of Biological, Earth and Environmental Sciences, Faculty of Science, The University of New South Wales, Kensington, NSW 2052, Australia

2 – Climate Change Cluster, University of Technology Sydney, Broadway, New South Wales, Australia

3 – Universidade Federal do Rio de Janeiro, Instituto de Biologia, Departamento de Microbiologia Paulo de Goes, LEMM Laboratory, Rio de Janeiro, Brazil

4 – Division of Biological and Environmental Science and Engineering (BESE), King Abdullah University of Science and Technology, Biological and Environmental Science and Engineering Division, Thuwal, Saudi Arabia

† – These authors have contributed equally as second authors

^*^Corresponding authors

MSc. Alessandro do Nascimento Garritano

Centre for Marine Science and Innovation

School of Biological, Earth and Environmental Sciences

The University of New South Wales, NSW 2052, Australia

Email: ale@garritano.com.br

Prof. Torsten Thomas

Centre for Marine Science and Innovation

School of Biological, Earth and Environmental Sciences

The University of New South Wales, NSW 2052, Australia

Ph + 61 (0) 2 938 53467

Email: [t.thomas@unsw.edu.au](mailto:t.thomas@unsw.edu.au)

**Keywords:** hexactinellid, symbiosis, flux-balance analysis, metagenome-assembled genomes, metatranscriptomics, metabolic interactions

**Running title: Simple Porifera holobiont reveals complex interactions between the host, an archaeon, a bacterium, and a phage**

Conflict of Interest: The authors declare no conflict of interest


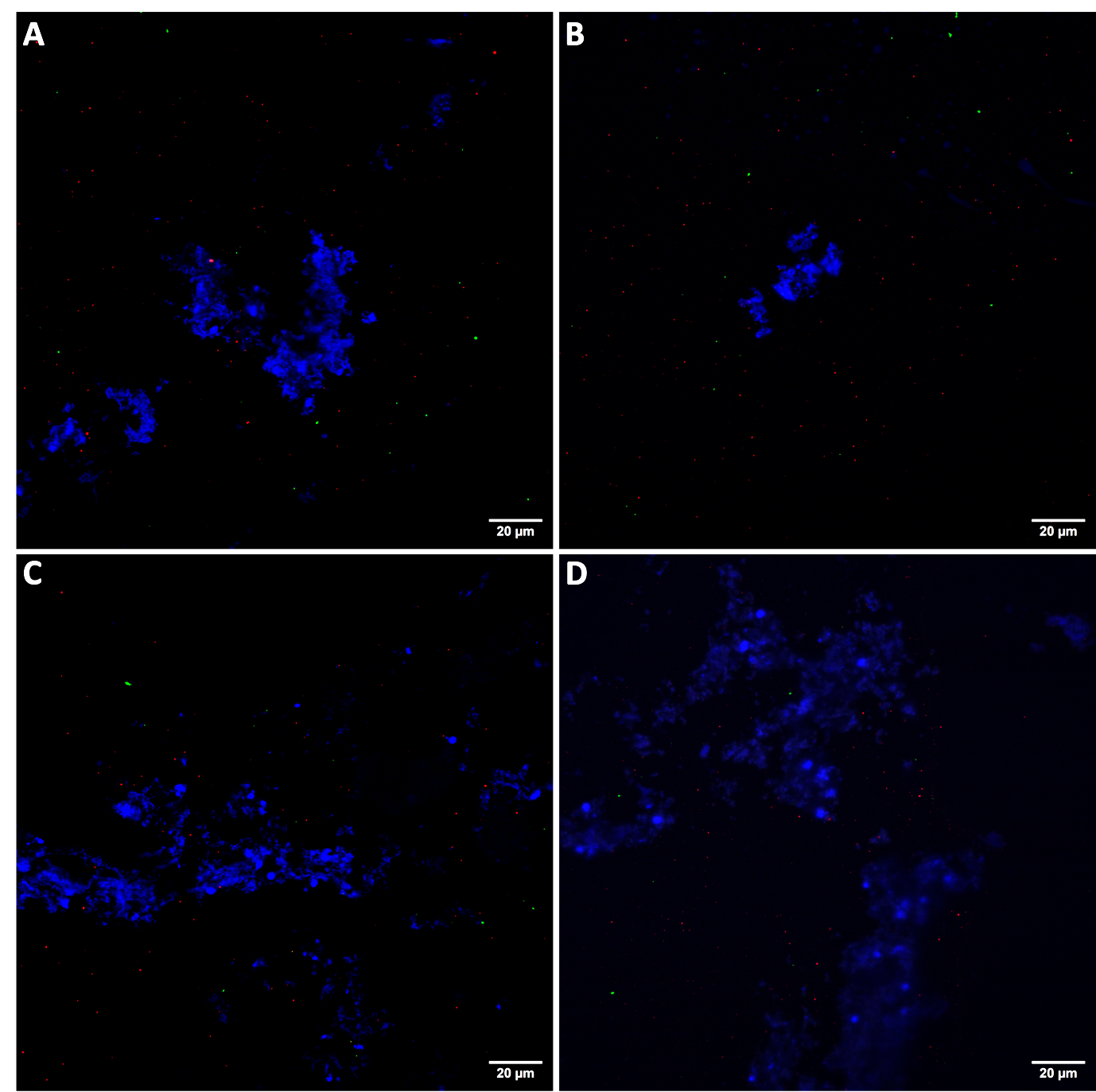


**Supplementary Figure 1:** FISH-labelled archaeal (red) and bacterial cells (green) as well as DAPI-stained DNA (bright blue) and sponge autofluorescence (faint blue). All images are 201.64 µm X 201.64 µm. Images **A, B, C** and **D** have 196, 263, 93 and 117 archaeal cells, respectively, while 21, 28, 18 and 12 bacterial cells, and 10, 3, 15, 20 sponge nuclei respectively, were found. Archaea therefore represent 90.32, 90.38, 83.78 and 90.70%, respectively, and bacteria represent 9.68, 9.62, 16.21 and 9.30% of all detected microbial cells.

**
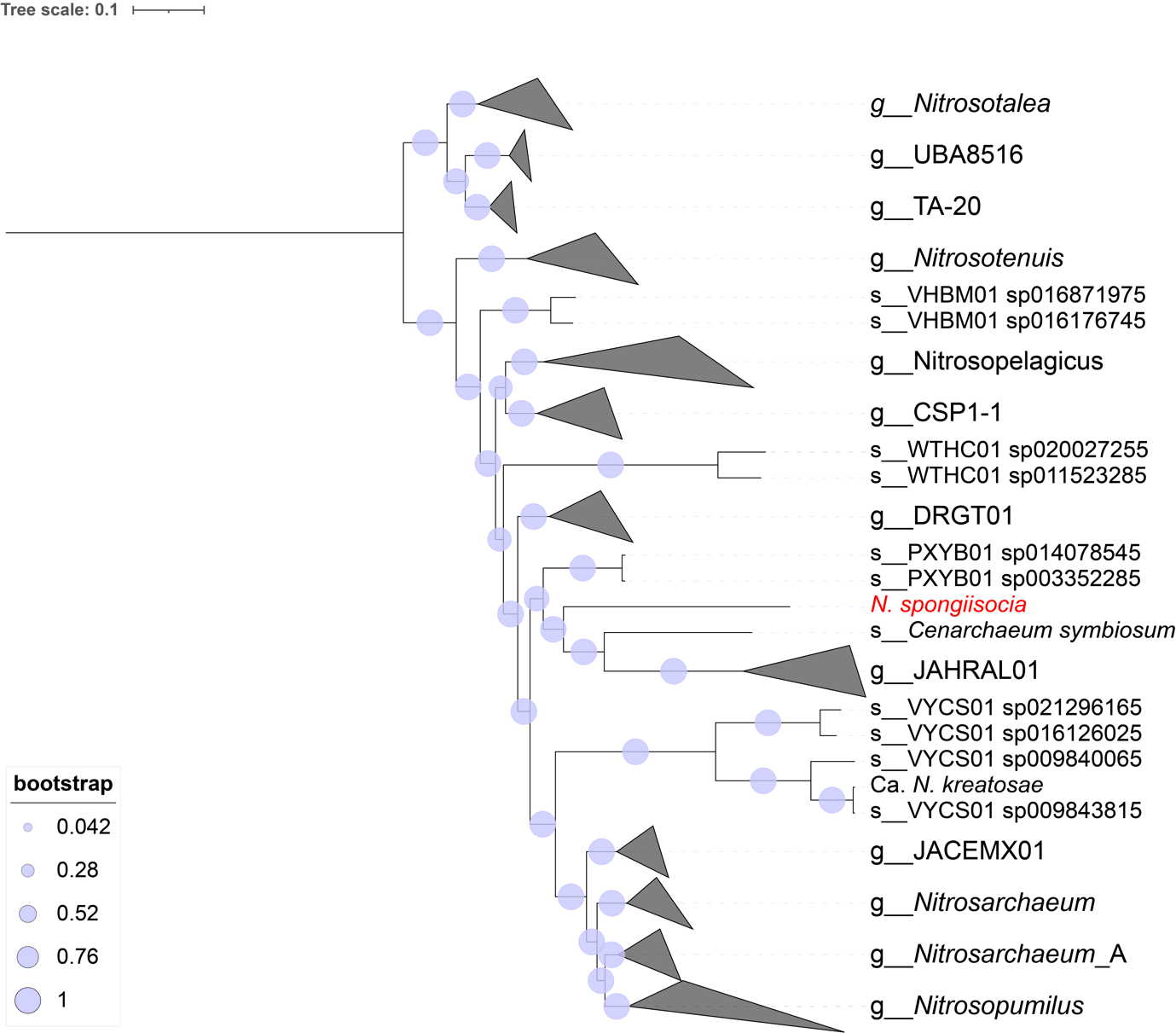
Supplementary Figure 2:** Phylogenetic tree for *Nitrosoabyssus spongiisocia* (red) and reference genomes from the GTDB database plus the recently described sponge-associated Ca. *Nitrosokoinonia kreatosae ^1^*. The tree was rooted using *Nitrososphaera viennensis* as an outgroup (not shown).


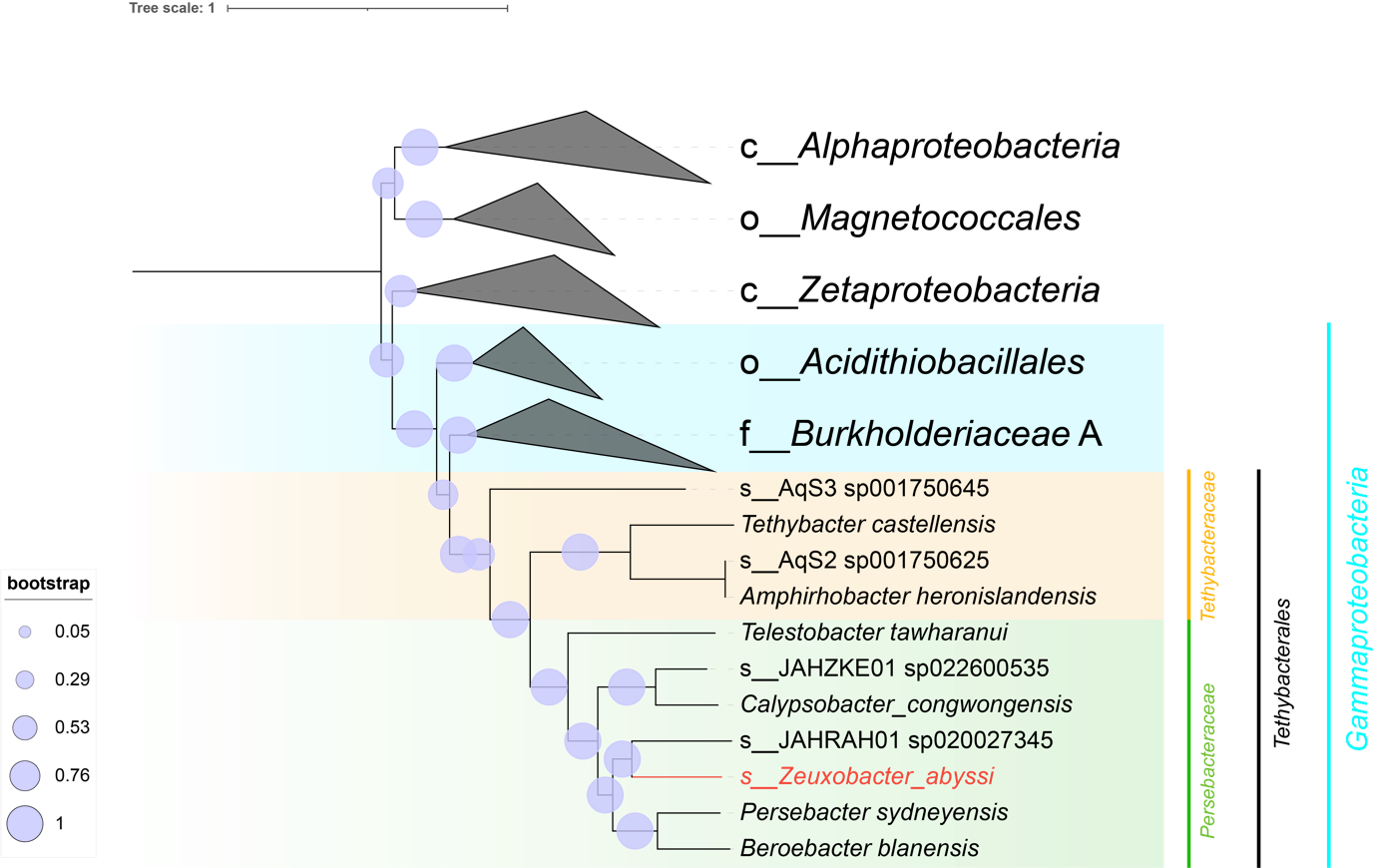


**Supplementary Figure 3:** Phylogenetic tree for *Z. abyssi* (red) and reference genomes from the GTDB database. The tree was rooted using *Sulfidibacter corallicola* (phylum *Acidobacteriota*) as an outgroup (not shown).

**
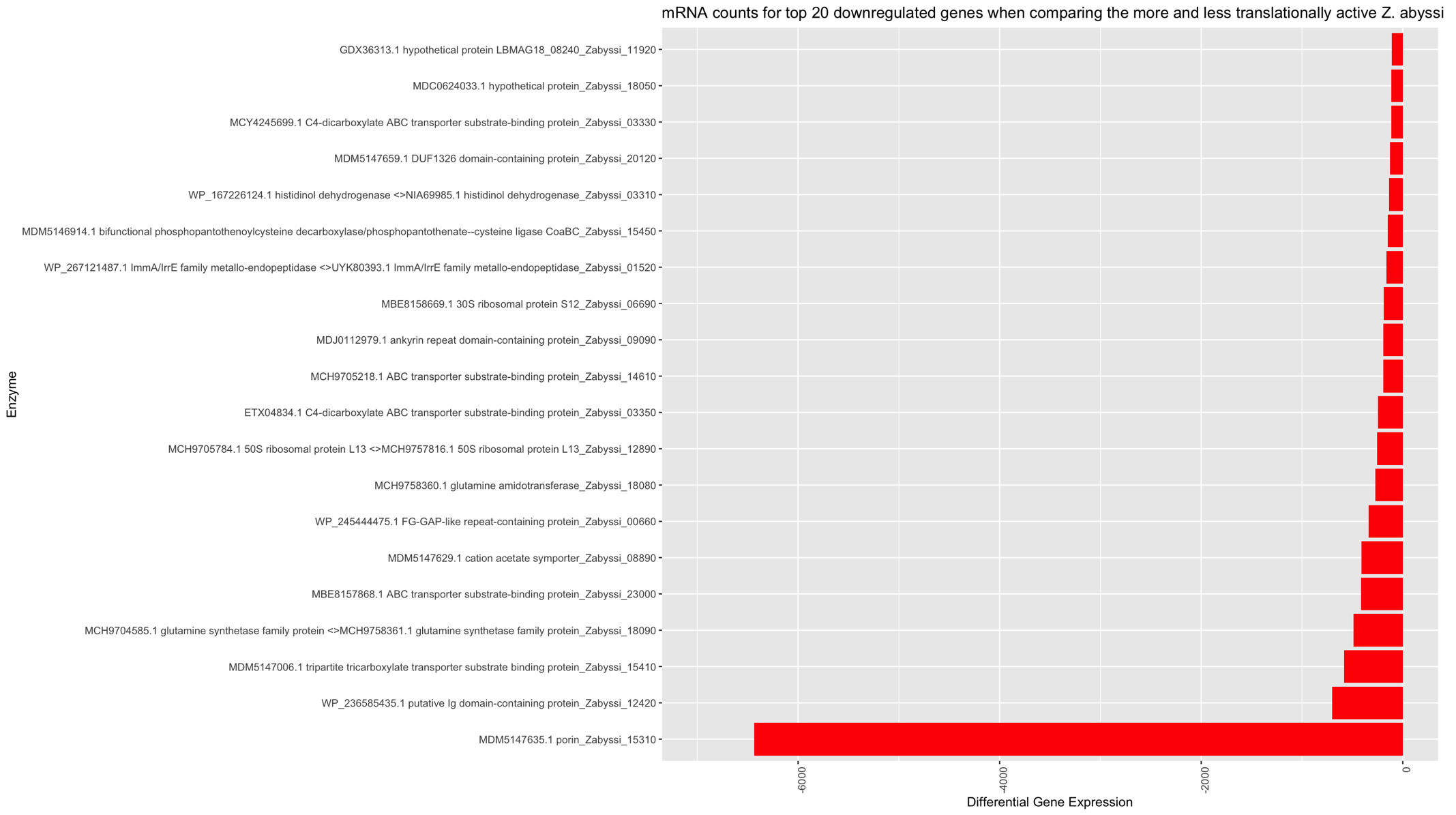
**

**Supplementary Figure 4:** Differential gene expression between samples with the more gene expression (S29 and S65) when compared to samples with the less gene expression (S44, S45, S70 and S71). Only genes with at least 10-fold expression changes were considered for the analysis and only the ones with top 20 greatest differences of the average of mRNA counts of the samples corresponding to the more versus the less expressing state are show. For a complete list see Supplementary Table 8.

**
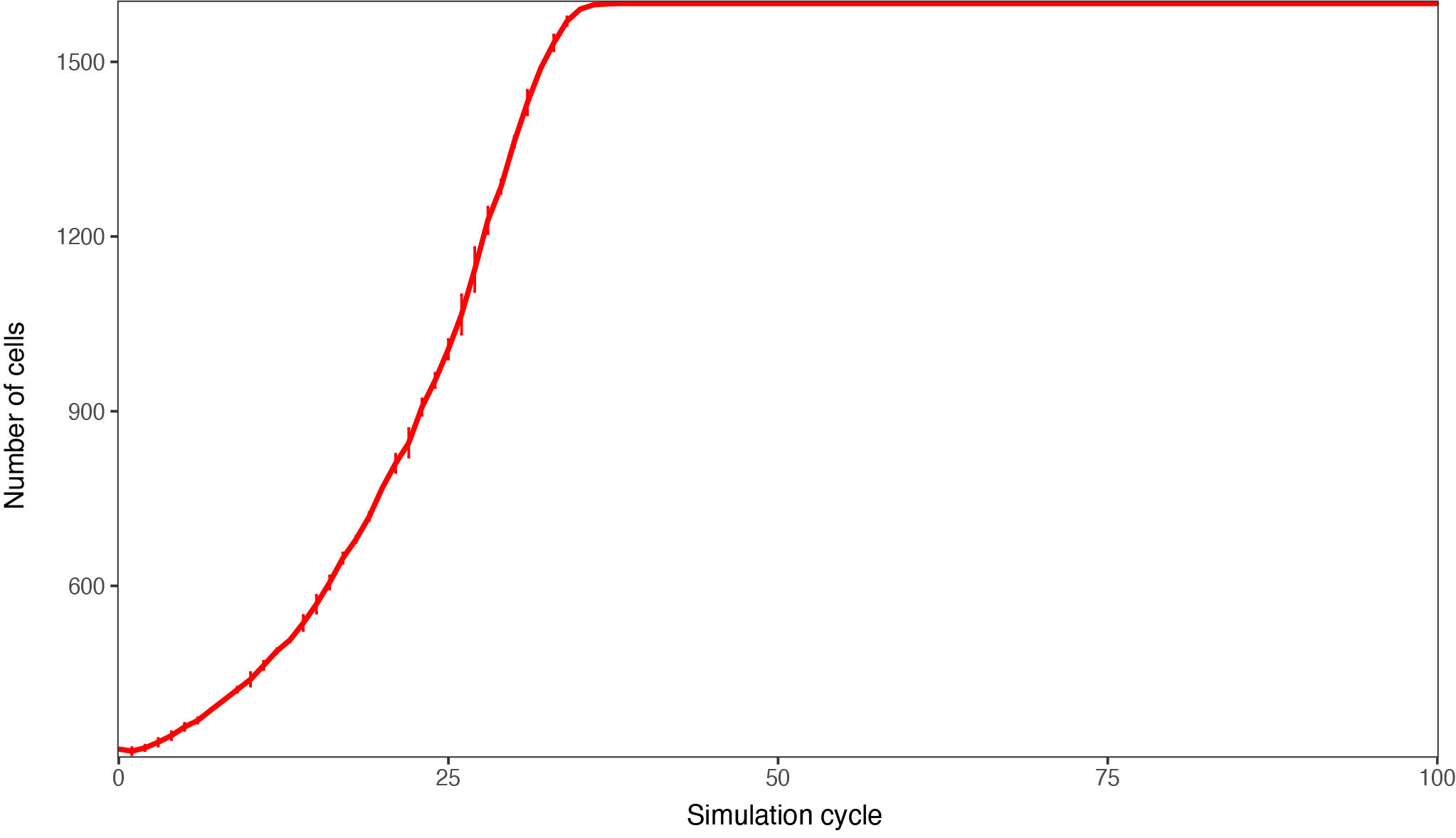
Supplementary Figure 5:** Growth simulation of *N. spongiisocia* over 100 cycles in a seawater medium without organic carbon sources (medium composition can be found in Supplementary Table 8).

**Supplementary Figure 6:** Average biomass increase of the metabolic model of *N. spongiisocia* over five simulation cycles under different ammonium concentrations. Blue dashed line shows the ammonium concentration where maximum growth rates were obtained under the medium condition shown in Supplementary Table 8. Cobalt then became limiting at maximum growth . Also, the metabolic model of *N. spongiisocia* only grew at a fraction of its maximum growth rate under the ammonium concentration found in the deep-sea site (magenta dashed line; and Supplementary Table 9).

**Supplementary Figure 7:** Growth simulation of *Z. abyssi* over 100 cycles in a seawater-based medium with taurine, DMSP, creatine, choline, betaine and vitamin B12 (see Supplementary Table 8). Vitamin B_12_ was removed at the 10^th^ cycle, which lead to *Z. abyssi* becoming extinct.

**Supplementary Figure 8:** Ordination plot representing the overall metabolic flux profile for each simulation cycle. The first stable flux profile is found between cycles 20 and 50 and is represented in green. The second stable metabolic profile is found between cycles 80 and 155 and is shown in cyan. The third stable metabolic profile is found between cycles 180 and 240 and is represented in purple. All the other flux profiles are represented in red.

**
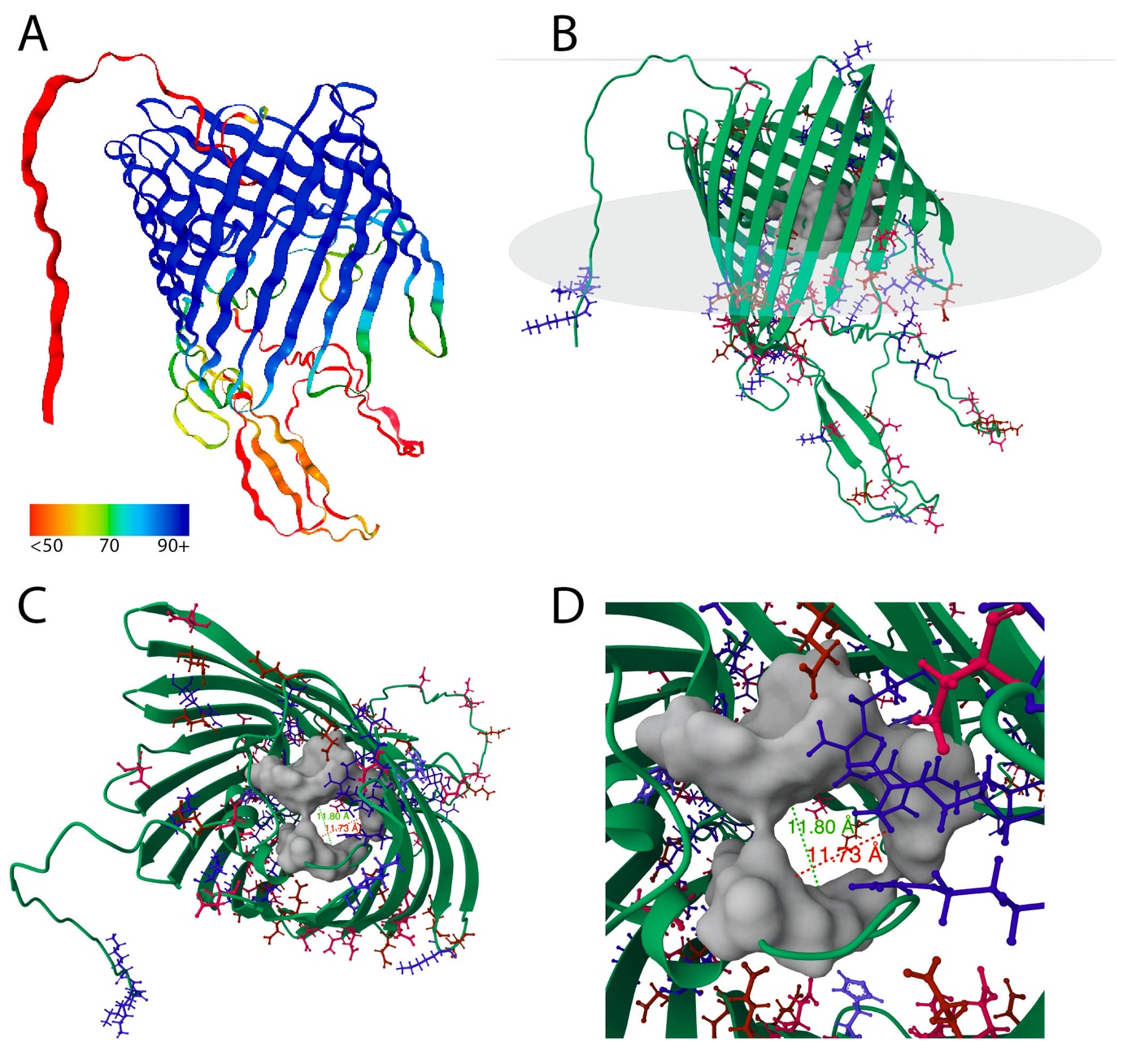
Supplementary Figure 9:** Structural analysis of the porin encoded by gene Zabyssi_15310. A: Alphafold2 structural prediction of the porin protein. Heat bar represents the confidence assigned to each region. The 16-stranded ß-barrel has a confidence value higher than 90% and the pore region has an approximate confidence value of 80%. B: Frontal view of the porin structure, with the pore region represented in grey, positively charged amino acids represented in blue and negatively charged amino acids represented in red. The grey discs represent the bacterial cell wall C: Top view of the porin structure, highlighting the pore in grey. D: Zoom of the pore structure, showing the diameter measurements of the pore as well as the high amount of positively charged amino acids close to it.

**Prediction of porin function**

Structural analysis of the porin encoded by gene Zabyssi_15310 using Alphafold ^2^ revealed strong similarity to the porin Omp32, which is known to have inside its pore several arginine residues that create a strong positive potential to allow for the binding of divalent anions ^3^. Furthermore, this type of pore has been found in other organisms as a selective transporter for divalent anions, including dicarboxylates ^3^. The pore is also known to have a particularly narrow constriction zone with an approximate diameter of 11 Å, providing not only a charge-based selection mechanism, but also a size-based selection mechanism ^3,4^. Amino acid residue analysis in this predicted protein fold revealed a pattern of positively charged amino acids in the interior of the pore (Supplementary Figure 10C), followed by a slightly wider constriction zone than the one found in Omp32 (Supplementary Figure 10D), of about 11.8 Å. Negatively charged amino acids seem to be concentrated in the region of the protein that is turned towards the cytosol of the cell, after the constriction zone, which we speculate would help the divalent anions to be released in the cytosol, providing an efficient transport from the extracellular environment into the cell.

**
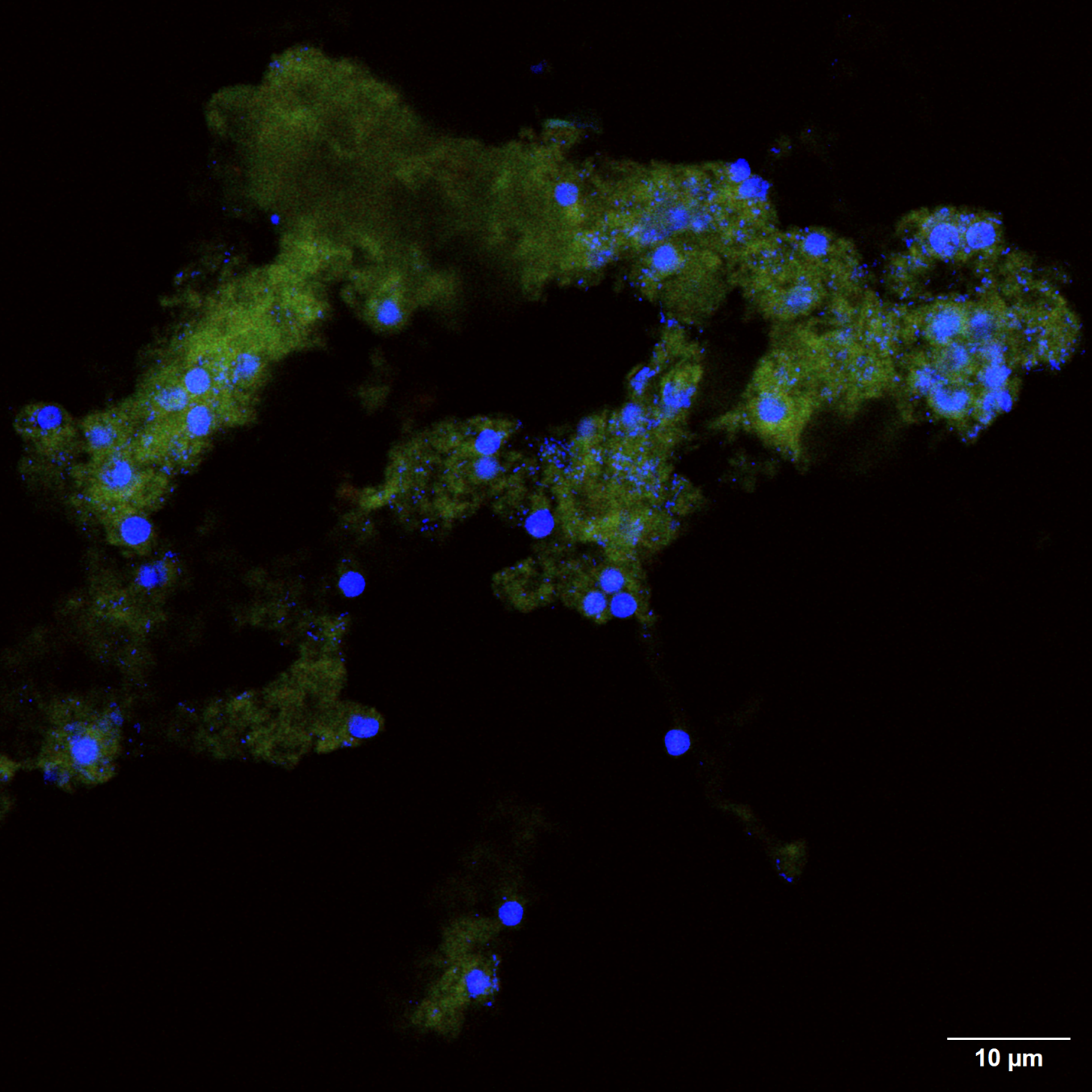
**

**Supplementary Figure 10:** Fluorescence *in situ* hybridization for an *A. beatrix* sample using the negative control probe NONEUB338 labelled with Cy5 (expected red signal). Sponge autofluorescence is shown in green and DNA is show in blue (DAPI). The absence of a red signal shows that DNA probes don’t bind unspecifically to the *A. beatrix* tissue.

**Etymology for *Nitrosoabyssus spongiisocia:***

**Syllabication**

Ni.tro.so.a.bys'sus

**Etymology**

**L. masc. adj.** *nitrosus*, nitrous; **L. fem. n.** *abyssus*, a bottomless pit; **N.L. fem. n.** *Nitrosoabyssus*, A nitrifying organisms from the depths

**Syllabication**

spon.gi.i.so'ci.a

**Etymology**

**L. fem. n.** *spongia*, sponge; **L. masc. n.** *socius*, companion; **N.L. masc. n.** *spongiisocia*, the companion of a sponge

**Etymology for *Zeuxobacter abyssi:***

**Syllabication**

*Zeuxoniibacter*

**Etymology**

**N.L. fem. n.***Zeuxo,* a water nymph in the Greek mythology and daughter of *Tethys;* **N.L. masc. n.** *bacter*, a short bacterium; **N.L. masc. n*.****Zeuxoniibacter,* a bacterium named after the water nymph *Zeuxo*

**Syllabication**

*abyssi*

**Etymology**

**N.L. gen. n*.****abyssi,* from the abyss, deep or bottomless pit; **N.L. gen. n*.****abyssi,* from the deep

**Etymology for *Nitrosopumivirus cobalaminus (*nov. Order. Iaravirales, nov. Fam. Anhangaviridae)**

**Syllabication**

*Iaravirales*

**Etymology**

**N.L. fem. n*.****Iara*, the water Goddess in the Tupi-Guarani (one of the first nation people in Brazil) mythology. N.L. masc. n*.* *virales*, a suffix for viral orders. **N.L. masc. n*.****Iaravirales,* a viral order named after the water Goddess Iara

**Syllabication**

*Anhangaviridae*

**Etymology**

***N.L. masc. n.****Anhanga*, the God of the abyss, the underworld in the Tupi-Guarani (one of the first nation people in Brazil) mythology. **N.L. masc. n.** *viridae*, a suffix for viral families. **N.L. masc. n*.****Anhangaviridae,* a viral family named after the abyss God Anhanga

**Syllabication**

*Nitrosopumivirus*

**Etymology**

**N.L. masc. n.***Nitrosopumi*, relative to the organisms of the archaeal family *Nitrosopumilaceae*. **N.L. masc. n.** *virus,* a suffix for viral genera. **N.L. masc. n*.****Nitrosopumivirus,* a viral genus involved that infects *Nitrosopumilacae*.

**Syllabication**

*cobalaminus*

**Etymology**

**N.L. masc. n.***cobalamin*, vitamin B12. **N.L. masc. n.** *cobalaminus*, related to vitamin B12.

**References:**

1. Glasl, B. *et al.* Co-occurring nitrifying symbiont lineages are vertically inherited and widespread in marine sponges. *ISME J* (2024) doi:10.1093/ismejo/wrae069.

2. Jumper, J. *et al.* Highly accurate protein structure prediction with AlphaFold. *Nature* **596**, 583–589 (2021).

3. Zeth, K., Diederichs, K., Welte, W. & Engelhardt, H. Crystal structure of Omp32, the anion-selective porin from Comamonas acidovorans, in complex with a periplasmic peptide at 2.1 Å resolution. *Structure* **8**, 981–992 (2000).

4. Zachariae, U., Klühspies, T., De, S., Engelhardt, H. & Zeth, K. High Resolution Crystal Structures and Molecular Dynamics Studies Reveal Substrate Binding in the Porin Omp32. *Journal of Biological Chemistry* **281**, 7413–7420 (2006).
